# Cost-effectiveness of steroid (methylprednisolone) injections versus anaesthetic alone for the treatment of Morton’s neuroma: economic evaluation alongside a randomised controlled trial (MortISE trial)

**DOI:** 10.1186/s13047-015-0064-y

**Published:** 2015-02-25

**Authors:** Rhiannon Tudor Edwards, Seow Tien Yeo, Daphne Russell, Colin E Thomson, Ian Beggs, J N Alastair Gibson, Diane McMillan, Denis J Martin, Ian T Russell

**Affiliations:** Bangor University, Centre for Health Economics and Medicines Evaluation (CHEME), School of Healthcare Sciences, College of Health and Behavioural Sciences (CoHaBS), Ardudwy Hall, Normal Site, Bangor, LL57 2PZ, Gwynedd UK; Swansea University, Singleton Park, Institute of Life Science 2, College of Medicine, Swansea, SA2 8PP UK; Health Sciences, Queen Margaret University, Queen Margaret University Drive, Edinburgh, EH21 6UU Scotland UK; The Royal Infirmary of Edinburgh, 51 Little France Crescent, Department of Radiology, Old Dalkeith Road, Edinburgh, EH16 4SA Scotland UK; Musculoskeletal Directorate, The Royal Infirmary of Edinburgh, 51 Little France Crescent, Old Dalkeith Road, Edinburgh, EH16 4SA Scotland UK; Teesside University, Health and Social Care Institute, Middlesbrough, TS1 3BA UK

**Keywords:** Morton’s neuroma, Interdigital plantar nerves, Methylprednisolone, Steroid injection, Foot health, Cost-effectiveness analysis, Cost-utility analysis, Quality-adjusted life years

## Abstract

**Background:**

Morton’s neuroma is a common foot condition affecting health-related quality of life. Though its management frequently includes steroid injections, evidence of cost-effectiveness is sparse. So, we aimed to evaluate whether steroid injection is cost-effective in treating Morton’s neuroma compared with anaesthetic injection alone.

**Methods:**

We undertook incremental cost-effectiveness and cost-utility analyses from the perspective of the National Health Service, alongside a patient-blinded pragmatic randomised trial in hospital-based orthopaedic outpatient clinics in Edinburgh, UK. Of the original randomised sample of 131 participants with Morton’s neuroma (including 67 controls), economic analysis focused on 109 (including 55 controls). Both groups received injections guided by ultrasound. We estimated the incremental cost per point improvement in the area under the curve of the Foot Health Thermometer (FHT-AUC) until three months after injection. We also conducted cost-utility analyses using European Quality of life-5 Dimensions–3 Levels (EQ-5D-3L), enhanced by the Foot Health Thermometer (FHT), to estimate utility and thus quality-adjusted life years (QALYs).

**Results:**

The unit cost of an ultrasound-guided steroid injection was £149. Over the three months of follow-up, the mean cost of National Health Service resources was £280 for intervention participants and £202 for control participants – a difference of £79 [bootstrapped 95% confidence interval (CI): £18 to £152]. The corresponding estimated incremental cost-effectiveness ratio was £32 per point improvement in the FHT-AUC (bootstrapped 95% CI: £7 to £100). If decision makers value improvement of one point at £100 (the upper limit of this CI), there is 97.5% probability that steroid injection is cost-effective. As EQ-5D-3L seems unresponsive to changes in foot health, we based secondary cost-utility analysis on the FHT-enhanced EQ-5D. This estimated the corresponding incremental cost-effectiveness ratio as £6,400 per QALY. Over the recommended UK threshold, ranging from £20,000 to £30,000 per QALY, there is 80%-85% probability that steroid injection is cost-effective.

**Conclusions:**

Steroid injections are effective and cost-effective in relieving foot pain measured by the FHT for three months. However, cost-utility analysis was initially inconclusive because the EQ-5D-3L is less responsive than the FHT to changes in foot health. By using the FHT to enhance the EQ-5D, we inferred that injections yield good value in cost per QALY.

**Trial registration:**

Current Controlled Trials ISRCTN13668166

## Background

Morton’s neuroma is the common descriptive term for a benign neural swelling of one of the interdigital plantar nerves. The condition most commonly affects the nerves in the second and third interspaces and is more common in middle-aged women [1,2]. Causal factors may include high heeled or ill-fitting shoes, high impact athletic activities such as jogging and direct trauma [3,4]. Symptoms from Morton’s neuroma may include paraesthesia, burning, numbness and pain [1,2,4]. The clinical and economic significance of Morton’s neuroma is that prolonged disabling foot pain can lead to limitations in daily activities and sickness absence [2]. Treatment options include insertion of insoles into footwear, steroid injections to manage foot pain, and eventual surgery [5-7].

Steroid injections are common second-line interventions but evidence of effectiveness and cost-effectiveness is sparse [2]. This paper presents the economic findings of a randomised trial of steroid injection in the treatment of Morton’s neuroma [8]. This trial found that in comparison with the control group, foot health in the corticosteroid group was significantly better at one and three months: at three months the mean difference was 14.1 points on the Foot Health Thermometer (95% confidence interval: 5.5 to 22.8 points; p = 0.002). Corticosteroid injections also improved pain, function and general health one and three months after injection. The size of the neuroma on ultrasound did not significantly influence the effect of treatment.

Since then there has been little change in clinical practice; steroid injections remain a second-line treatment for Morton’s neuroma. The current systematic review for the Cochrane Library [9] stresses the lack of clinical evidence for this common condition. Hence, our primary economic aim was to investigate the cost-effectiveness of steroid injection compared with anaesthetic injection alone in the treatment of Morton’s neuroma in reducing foot pain over three months.

## Methods

### Study design and interventions

The economic evaluation took place alongside the trial known as ‘Morton’s neuroma: Injected Steroids Effective?’ (MortISE) [Registration: http://www.controlled-trials.com/ISRCTN13668166], a patient-blinded pragmatic randomised trial designed to compare the effectiveness of steroid injections with anaesthetic injections in alleviating pain and other effects of Morton’s neuroma [8]. The trial included 131 patients with clinical symptoms of Morton’s neuroma; their mean age was 53 years, and 111 were female. We randomised participants between an intervention group receiving corticosteroid and anaesthetic injections [1 ml methylprednisolone (40 mg) and 1 ml 2% lignocaine] and a control group receiving anaesthetic alone (2 ml 1% lignocaine). We kept trial participants blind to the type of injection they received. Though we had intended also to keep the radiologist blind, this proved impractical. Nevertheless the radiologist played no other part in the study [8].

Participants completed measures at baseline before treatment and at follow-up clinics one and three months after randomisation. We chose the interval of one month to maximise clinical benefit and identify any adverse events following injection; and that of three months to estimate medium-term benefit. We collected the following outcome data for the trial: neuroma size, foot pain and disability by the Foot Health Thermometer (FHT) and the Manchester Foot Pain and Disability Schedule, pain by the Multidimensional Affect and Pain Survey and health-related quality of life by the European Quality of life-5 Dimensions–3 Levels (EQ-5D-3L). The primary outcome was participants’ assessment of their foot health by the FHT at three months after injection.

The Lothian Research Ethics Committee approved the study. All participants provided written informed consent before starting the study.

### Economic evaluation

We conducted an economic evaluation three months after randomisation of participants. Our aim was to evaluate steroid injections for the treatment of Morton’s neuroma. To be comprehensive we addressed both cost-effectiveness, which focuses on foot health, through the FHT, and cost-utility, which addresses health-related quality of life. For the latter, the National Institute for Health and Care Excellence (NICE) in the UK has recommended the use of quality-adjusted life years (QALYs) as the measure of health benefit for economic analysis as it allows comparisons across different clinical conditions, unlike condition-specific outcomes like the FHT [10].

As it is not easy to impute missing cost data, we based economic analysis of this trial on a smaller sample than the effectiveness analysis. Data for the costs of service use were incomplete for 10 participants in the intervention group and 12 participants in the control group, though we had included 13 of them (5 intervention and 8 control) in effectiveness analyses. We had full economic data and enough baseline and follow-up data to impute QALYs and the area under the curve of the Foot Health Thermometer (FHT-AUC) for 109 participants – 54 in the intervention group and 55 participants in the control group. This represented 89% of the 122 participants in the main effectiveness analysis [8].

### Measurement of costs

We examined costs from the perspective of the National Health Service [11,12]. We estimated direct primary and secondary care use from hospital records and participants’ self-reported client service receipt inventories [13] at one and three months. Research resource constraints prevented us from asking participants to complete client service receipt inventories at baseline. At one and three months the client service receipt inventories asked patients to recall all contacts with primary care, attendances at emergency departments, and inpatient stays. From hospital records we gathered data on steroid and anaesthetic injections, outpatient visits, surgery, radiological imaging, and laboratory tests and investigations. We derived unit costs of these service contacts in pounds Sterling from national sources [14,15] (Table 1).

**Table 1:** **Unit cost (£) and source of health service use in the UK** ^**a, b, c**^

**Health-care resource**	**Unit**	**Unit cost (£)** ^**a**^	**Details and source**
Primary care contacts, e.g. general practitioner, practice nurse.	Consultation	12.30 to 84.86	Costed by profession^d^
PAMs, e.g. physiotherapist, chiropodist, consultant radiologist	Consultation	12.30 to 51.65	Costed by profession^d^
Hospital outpatient clinic e.g. orthopaedic, opthalmologist	Consultation	103.31 to 110.69	Costed by specialty^d, e^
Hospital outpatient consultation with ultrasound scan	Consultation with scan	145.74	Cost including costs of consultant radiologist lasting 30mins, nurse lasting 30 minutes of client contact and an ultrasound scan^d,e^
Hospital outpatient consultation with no ultrasound scan	Consultation with no scan	63.34	Cost of consultation lasting 30mins^e^
Inpatient hospital stay	Procedure	154.96	Costed by procedure^e^
Accident and emergency	Consultation	28.29	Costed by consultation^e^
Steroid injection (1ml methylprednisolone (40 mg) and 1ml 2% Lignocaine)	Item	3.74	Costed by BNF entry^f^
Anaesthetic injection (2ml of 1% Lignocaine)	Item	0.25	Costed by BNF entry^f^

Legend: Table 1 Unit cost of health service use in UK pounds sterling (£)^a^, with source^b, c^.

PAMs: Professionals Allied to Medicines; BNF: British National Formulary.

^a^Cost year 2011/12.

^b^National Health Service costs including salary, employers’ costs, overheads and capital costs.

^c^Costs extracted from Curtis and Netten [11] and Department of Health [10] have been inflated from 2004/05 to 2011/12 using Hospital & Community Health Service inflation indices from Curtis [12].

^d^From Curtis and Netten [11].

^e^From Department of Health [10].

^f^From BNF [13].

We inflated these costs extracted from Department of Health [14] and Curtis and Netten [15] from 2004–5 to 2011–2 using Hospital & Community Health Service inflation indices [16]. We obtained costs of steroid and anaesthetic drugs from the British National Formulary version 52 [17] and inflated them to 2011–2 prices. During the three-month follow-up period, three participants (two in the intervention group and one in the control group) underwent surgical procedures for gastroenterological and gynaecological conditions. As these inpatient costs did not relate to foot health, we did not include them in our analysis.

### Measurement of effectiveness

Our primary analysis was a cost-effectiveness analysis [10,18] using FHT-AUC, the area under the curve of FHT scores, to measure the outcome of the trial. The FHT is a validated visual analogue scale similar to the EQ-5D-3L thermometer with 0 representing worst possible foot health and 100 best possible foot health [19]. We corrected all effects for differences in baseline, thus improving the accuracy of estimated effects.

We undertook a cost-utility analysis using QALYs as the measure of effect. We estimated participant utilities by administering the EQ-5D-3L instrument [20] at baseline, one month and three months; combined them using the area under the curve method to calculate QALY gains over the three month study period; and corrected for baseline EQ-5D-3L. We estimated the cost per QALY gain by dividing differences in cost by difference in QALYs and compared these with the thresholds recommended by NICE in the UK [10]. We did not discount costs or effects as the follow-up period was less than one year.

To quantify the uncertainty around the estimated incremental cost-effectiveness ratios, for both cost-effectiveness and cost-utility analyses, we ran a simulation of 5000 non-parametric bootstrapped iterations using MS Excel 2007. We used these to estimate confidence intervals (CIs) for incremental costs and incremental cost-effectiveness ratios and to construct a cost-effectiveness plane – a scatter plot of the joint distribution of incremental costs and effects – and a cost-effectiveness acceptability curve [21]. The cost-effectiveness acceptability curve displays the probability that an intervention is more cost-effective than the alternative across a range of thresholds of willingness to pay for a QALY [22,23].

### Sensitivity analyses

To assess how dependent on our original assumptions our findings are, we undertook two forms of sensitivity analysis. Firstly, we recognised that the MortISE trial had adopted as its primary measure of benefit – the FHT, a Patient-Reported Outcome Measure with evidence of responsiveness to change [8]. In contrast, for our cost-utility analysis alongside MortISE we chose as primary measure of benefit – the EQ-5D-3L, a PROM with a strong theoretical basis for its economic validity [24,25]. However, as there are concerns that the use of three-point scales by EQ-5D-3L reduces its responsiveness to change [26,27], we regressed the EQ-5D-3L data from the MortISE participants as the dependent variable on their FHT data as the independent variable, with allocated treatment as the covariate. The resulting regression equation has two complementary functions: it converts participants’ responses to the FHT into utilities on the original EQ-5D-3L scale, and it uses the greater discrimination achieved by the FHT to fill gaps in the simplistic three-point scales that characterised the original EQ-5D-3L. Though the custodians of the EQ-5D-3L have recently sought to improve responsiveness by expanding scales to five points in the EQ-5D-5L [28], the fact that we conceived MortISE before then stimulated us to find another method of strengthening the EQ-5D-3L, which we call the FHT-enhanced EQ-5D.

Secondly, we recognised that the costing of steroid injections depended on the design of MortISE, which performed ultrasound scans on all trial participants; in the intervention group to guide the steroid injections, and in the control group to assess the neuroma and need for surgery. Though this design aimed to deliver best practice to the control group, it also had the explanatory aim [29] of equalising the ‘placebo effect’ between groups; because both groups received ultrasound scans, we could blind them to whether their injection contained steroid or anaesthetic. For our second sensitivity analysis we made the more pragmatic assumption [29] that control participants could attend the outpatient clinic for review and injection without receiving an ultrasound scan. If so, the unit cost of their hospital appointment, previously £145.74 including ultrasound scan, would fall to £63.34 without ultrasound scan. We then added the unit cost of anaesthetic injection (£0.25) to yield a total cost of £63.59 for hospital appointment with anaesthetic injection but no ultrasound scan.

### Computing software

We analysed data in SPSS version 16.0 (SPSS Inc., Chicago II, USA) and MS Excel 2007 (Microsoft Corporation, Redmond, Washington DC, USA).

## Results

### Baseline demographic characteristics of study participants

Table 2 summarises the demographic characteristics of participants in the intervention and control groups at baseline. As in the main effectiveness paper [8], demographic variables were similar across intervention and control groups.

**Table 2:** **Baseline demographic characteristics of the participating patients with Morton’s neuroma** ^**a**^

	**Control group (n = 55)**	**Steroid group (n = 54)**
**Gender**		
Male	9 (16%)	10 (19%)
Female	46 (84%)	44 (81%)
**Age (year)**		
Mean (SD); range	52.6 (12.3); 26-76	54.3 (12.2); 28-79
**Current smoker**		
Smoker	6 (11%)	6 (11%)
Ex-smoker	17 (31%)	17 (32%)
Non-smoker	32 (58%)	30 (56%)
Missing	0 (0%)	1 (2%)
**Employment**		
In employment	34 (62%)	33 (61%)
Retired	12 (22%)	12 (22%)
Housework	7 (13%)	5 (9%)
Other	2 (4%)	3 (6%)
Missing	0 (0%)	1 (2%)
**Education**		
Continued after the minimum school leaving age^b^		
Yes	31 (56%)	32 (60%)
No	24 (44%)	21 (39%)
Missing	0 (0%)	1 (2%)
Degree or equivalent^b^		
Yes	18 (33%)	19 (35%)
No	37 (67%)	35 (65%)
Missing	0 (0%)	0 (0%)
**Body Mass Index (BMI) (kg/m** ^**2**^ **)**		
Mean (SD); range	27.7 (4.2); 21.3-40.7	27.7 (5.4); 21.1-52.1

^a^Figures are numbers (percentages) of the patients unless stated otherwise. Percentages may not add to 100 due to rounding.

^b^These two categories are not mutually exclusive.

### Effectiveness

The effectiveness results for our economic sample of 109 participants were similar to those for the slightly larger sample in the main effectiveness paper [8]: steroid injections significantly improved foot pain (by more than ten points on the FHT) at both one month (p = 0.006) and three months (p = 0.013); but had no significant effect on health-related quality of life as measured by EQ-5D-3L. Combining the results for each of the study time points (baseline, one month and three months) into areas under the curves and correcting for baseline, the intervention group had significantly better FHT-AUC than the control group, with a mean difference of 2.472 points (95% CI: 0.986 to 3.958; p = 0.001). However, the mean QALY gain did not differ significantly between groups; the estimated improvement in QALY from using steroid injections was only 0.0038 (95% CI: −0.0146 to 0.0221, p = 0.68) [Table 3], which is 1.4 quality-adjusted life days.

**Table 3:** **Foot health thermometer scores and EQ-5D-3L utility index at baseline, 1 and 3 months**

**Outcome measure** ^**a**^	**(Control n = 55, Steroid n = 54)**				
	**Mean (SD)**		**Mean adjusted for the baseline (SE)**		**Estimated difference** ^**b**^ **[95% CI] significance**
	**Control**	**Steroid**	**Control**	**Steroid**	
**EQ-5D-3L utility index**					
Baseline	0.5831 (0.2947)	0.5346 (0.3023)	Not applicable	Not applicable	Not applicable
1 month	0.6213 (0.2523)	0.6129 (0.2717)	0.6178 (0.0351)	0.6164 (0.0354)	−0.0014 (−0.0976, 0.1004) p = 0.98
3 months	0.5944 (0.2852)	0.6226 (0.2771)	0.5930 (0.0388)	0.6241 (0.0388)	0.0311 (−0.0780, 0.1403) p = 0.57
**QALY**	0.1510 (0.0522)	0.1506 (0.0511)	0.1473 (0.0062)	0.1511 (0.0068)	0.0038 (−0.0146, 0.0221) p = 0.68
**Foot health thermometer scores**					
Baseline	48.17 (23.68)	45.23 (21.22)	Not applicable	Not applicable	Not applicable
1 month	49.30 (24.21)	60.28 (22.01)	48.49 (3.07)	60.81 (3.10)	**12.32 (3.66, 20.98) p = 0.006**
3 months	53.06 (26.77)	64.26 (22.05)	52.77 (3.36)	64.69 (3.32)	**11.92 (2.54, 21.29) p = 0.013**
**FHT-AUC**	12.469 (5.061)	14.738 (4.038)	12.276 (0.504)	14.748 (0.556)	**2.472 (0.986, 3.958) p = 0.001**

^a^Imputed where necessary.

^b^In the final column, positive differences represent a better outcome for participants in the steroid group, and results in bold type are significant at 5% level.

### Frequency and cost of steroid and anaesthetic injections

We estimated the costs of an outpatient visit for ultrasound-guided steroid injection as £149.48. This comprised £3.74 for the steroid injection and £145.74 for the outpatient appointment, including 30 minutes of consultant and nurse time, and an ultrasound scan. All intervention participants received one ultrasound-guided steroid injection at baseline.

We estimated the costs of an outpatient visit for an ultrasound-guided anaesthetic injection as £145.99. This comprised £0.25 for the anaesthetic injection and the same £145.74 for the outpatient appointment. All 55 control participants received one anaesthetic injection at baseline but two also received a steroid injection at follow up for the pain they were still experiencing (Table 4).

**Table 4:** **Mean frequency of health service use over three month follow-up period**

***Count; mean, median, (min, max)***	**Control (n = 55)**	**Steroid (n = 54)**
**NHS Secondary Sector:**		
**Injection:**		
Lignocaine only	55; 1.00, 1.00 (1, 1)	0; 0.00, 0.00 (0, 0)
Lignocaine & steroid	2; 0.04, 0.00 (0, 1)	54; 1.00, 1.00 (1, 1)
**Outpatient consultation with ultrasound scan**	55; 1.00, 1.00 (1, 1)	54; 1.00, 1.00 (1, 1)
**Outpatient follow-up visits:**		
Return visits	4; 0.07, 0.00 (0, 1)	6; 0.11, 0.00 (0, 1)
**Inpatient:**		
Inpatient hospital stay	1; 0.02, 0.00 (0, 1)	23; 0.43, 0.00 (0, 6)
**Accident and emergency**	4; 0.07, 0.00 (0, 2)	5; 0.09, 0.00 (0, 2)
**NHS Primary Care Sector:**		
**GP consultations**	54; 0.98, 1.00 (0, 4)	73; 1.35, 1.00 (0, 12)
**Other health care practitioner consultations**	29; 0.53, 0.00 (0, 9)	35; 0.65, 0.00 (0, 9)

NHS: National Health Service.

### Frequency and cost of service use by participants

Tables 4 and 5 show the frequency and cost of contacts with National Health Service primary and secondary care by participants in intervention and control groups. Table 5 shows mean costs of all services received by intervention and control participants over the three month follow-up period. These included ultrasound-guided injections, outpatient visits and primary care consultations.

**Table 5:** **Mean cost of health service use (£) over three month follow-up period**

**NHS Secondary Sector:**	**Control n = 55 mean (SD) in £**	**Steroid n = 54 mean (SD) in £**	**Mean difference in £ (95% CI bootstrapped)**
**Injection:**			
Lignocaine only	0.25 (0.00)	0.00 (0.00)	−0.25 (−)
Lignocaine & steroid	0.14 (0.71)	3.74 (0.00)	3.60 (−)
**Outpatient consultation with ultrasound scan**	145.74 (0.00)	145.74 (0.00)	0.00 (−)
**Outpatient follow-up visits:**			
Return visits	4.61 (16.60)	7.04 (20.09)	2.43 (−4.52, 9.43)
**Inpatient:**			
Inpatient hospital stay	2.82 (20.90)	66.00 (220.39)	63.19 (11.64, 126.32) *
**Accident and emergency**	2.06 (9.20)	2.62 (9.93)	0.56 (−3.05, 4.22)
**Secondary care costs**	**155.61 (32.85)**	**225.14 (222.19)**	69.53 (18.47, 148.49) *
**NHS Primary Care Sector:**			
**GP consultations**	31.57 (35.77)	39.95 (56.52)	8.37 (−8.49, 26.92)
**Other health care practitioner consultations**	14.51 (43.06)	15.28 (40.55)	0.77 (−15.10, 16.58)
**Primary care costs**	**46.09 (55.89)**	**55.23 (72.64)**	**9.14 (−14.68, 33.91)**
**Total costs**	**201.69 (67.74)**	**280.37 (246.74)**	78.67 (18.25, 152.34) *

NHS: National Health Service.

*Difference is significant at 5% level.

Table 5 shows mean cost differences and bootstrapped 95% CIs. The mean total cost per participant was £280.37 for the intervention group and £201.69 for the control group. The difference in mean cost between the intervention and control groups was £78.67 (bootstrapped 95% CI: £18.25 to £152.34).

### Primary cost–effectiveness analysis

The incremental cost–effectiveness ratio was £31.83 per point improvement in FHT-AUC (bootstrapped 95% CI from £6.79 to £99.94) – the result of dividing the difference in mean cost between intervention and control groups (£280.37 - £201.69 = £78.68; Table 5) by the difference in mean FHT-AUC between the two groups (14.748 - 12.276 = 2.472; Table 3).

### Incorporating uncertainty

**Figure 1:**
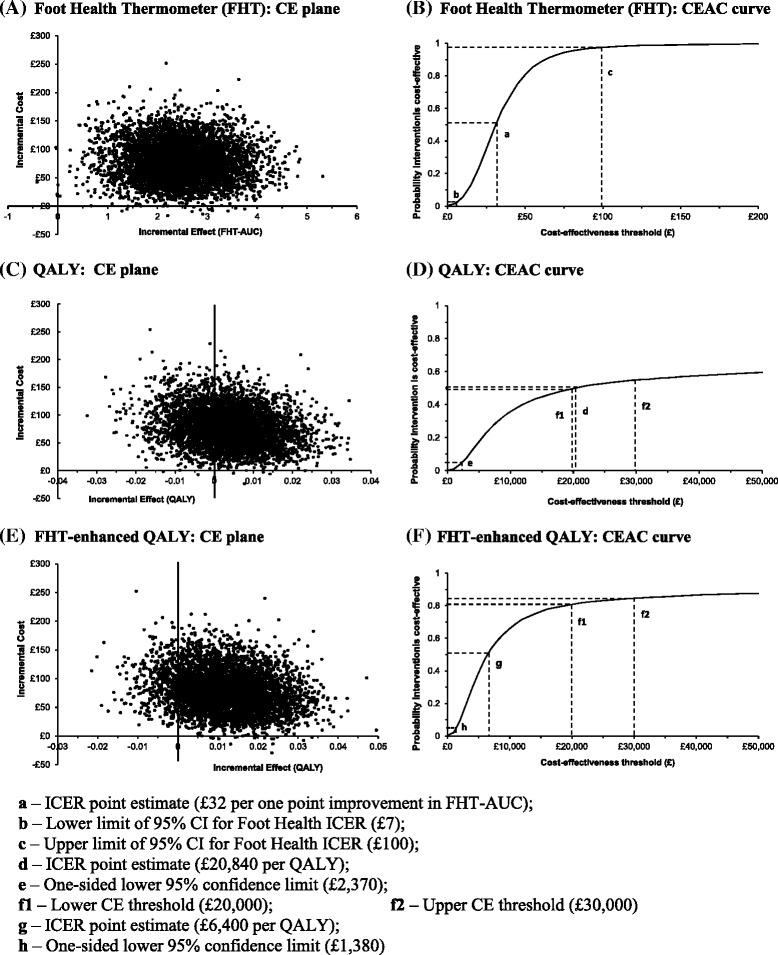
**Cost-effectiveness planes with 5,000 bootstrapped incremental cost-effectiveness ratio estimates for MortISE economic evaluation.** Cost-effectiveness planes for Foot Health Thermometer **(A)**, QALY **(C)** and FHT-enhanced QALY – 1st sensitivity analysis **(E)**; and cost-effectiveness acceptability curves at three months for Foot Health Thermometer **(B)**, QALY **(D)** and FHT-enhanced QALY – 1st sensitivity analysis **(F)**.

To assess uncertainty around incremental cost-effectiveness ratio estimates, we ran 5,000 bootstrap replications; Figure 1A shows the corresponding cost-effectiveness plane. Most points (99.54%) fall within the North East quadrant, where the intervention is both more costly and more effective than the control group; 19 bootstrapped replications (0.38%) fall in the South East quadrant (less costly, more effective) and the remaining four bootstrapped replications (0.08%) fall in the North West quadrant (more costly, less effective). Figure 1B shows the probability that the intervention is cost-effective for a range of cost thresholds. At the cost threshold of £100 for an improvement of one point in FHT-AUC, there is 97.5% probability that steroid injection is cost-effective.

### Cost-utility analysis

The mean health gain, expressed as a difference in mean QALYs between intervention and control groups over the three month follow-up period, was 0.0038 years, which is 1.4 quality-adjusted life days. We estimated the corresponding incremental cost-effectiveness ratio as £20,840 per QALY, by dividing the difference in mean cost of £78.68 between intervention and control groups (viz £280.37 - £201.69) by the difference in mean QALY of 0.0038 between the two groups (viz 0.1511 - 0.1473).

We performed 5000 bootstrapped replications to generate a cost-effectiveness plane and a cost-utility acceptability curve. Figure 1C shows the cost-effectiveness plane with 3307 points (66.14%) in the North East quadrant, 1686 (33.72%) in the North West quadrant, 2 (0.04%) in the South West quadrant and 5 (0.10%) in the South East quadrant. Figure 1D is the corresponding cost-utility acceptability curve. Because 33.72% of the bootstrapped estimates fall in the North West quadrant, where the intervention is more costly and less effective: this curve never reaches 70%; and estimation of an upper limit for a two-sided 95% CI is not possible. Therefore, only the bootstrapped one-sided 95% lower confidence limit of £2,370 appears on the cost-effectiveness acceptability curve (Figure 1D). In the NICE threshold range of £20,000 to £30,000 per QALY [10], the probability that steroid injection is cost-effective lies between 49.7% and 54.9%.

### Sensitivity analyses

Firstly, we replaced the EQ-5D by the FHT-enhanced EQ-5D, and bootstrapped another 5000 replications to produce a third pair of cost-effectiveness plane and cost-effectiveness acceptability curve. Figure 1E shows 4553 points (91.06%) in the North East quadrant, 431 (8.62%) in the North West quadrant, 16 (0.32%) in the South East quadrant, but none at all in the South West quadrant. Figure 1F presents the corresponding cost-utility acceptability curve. Again, the curve never reaches 1 because more than 125 (2.5%) of bootstrapped estimates fall in the North West quadrant, and the cost-effectiveness acceptability curve shows only the lower confidence limit of £1,380 (Figure 1F). The probability that steroid injection is cost-effective now lies between 80.8% and 84.6% over the NICE threshold range of £20,000 to £30,000 per QALY.

**Figure 2:**
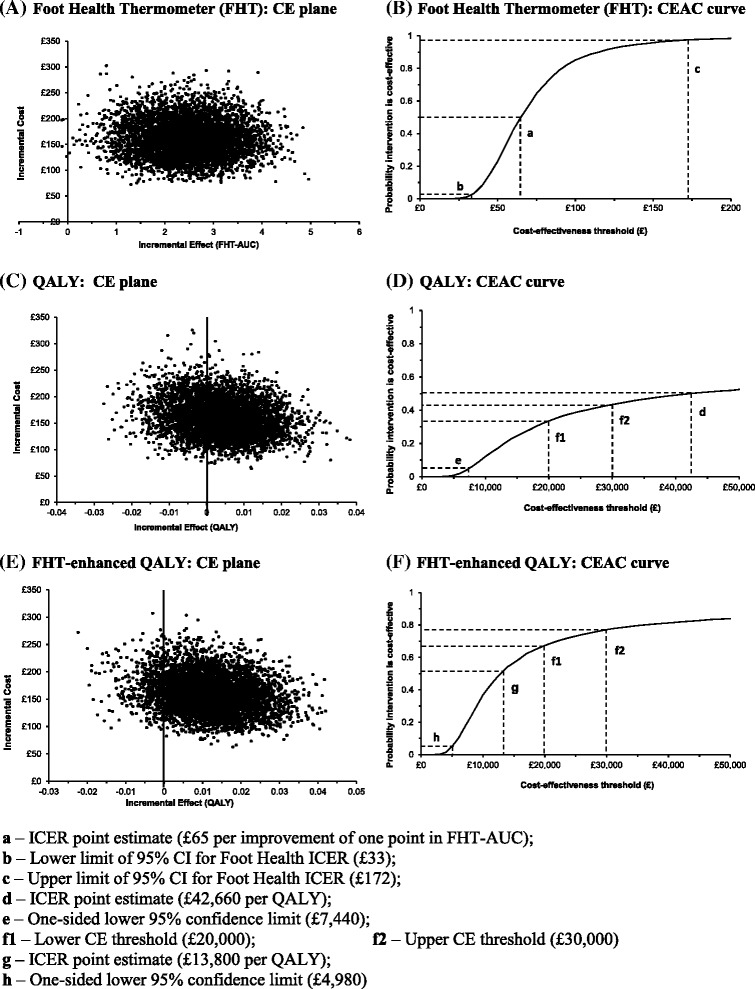
**Sensitivity analysis: cost-effectiveness planes with 5,000 bootstrapped incremental cost-effectiveness ratio estimates for MortISE economic evaluation.** 2nd sensitivity analysis: cost-effectiveness planes for Foot Health Thermometer **(A)**, QALY **(C)** and FHT-enhanced QALY **(E)**; and cost-effectiveness acceptability curves at three months for Foot Health Thermometer **(B)**, QALY **(D)** and FHT-enhanced QALY **(F)**.

Secondly, we repeated the bootstrap and recalculated the cost-effectiveness acceptability curve and incremental cost-effectiveness ratio point estimate as if patients in the control group had attended for an outpatient appointment and received an injection, but no ultrasound scan. In this scenario the mean cost for the intervention group remained unchanged, while the mean cost for the control group fell from £201.69 (hospital outpatient visit with ultrasound-guided injection) to £119.29 (hospital outpatient visit with injection but no ultrasound guidance scan). Thus, the incremental cost-effectiveness ratio rose from £31.83 to £65.16 per point improvement on the FHT-AUC (difference in mean total costs, £161.07, divided by difference in mean FHT-AUC, 2.472), with a bootstrapped 95% CI from £33.47 to £172.49. From the cost-effectiveness acceptability curve, the probability that steroid injection is cost-effective decreased to 85.2% at the cost threshold of £100 per point improvement in FHT-AUC (Figure 2B), compared with 97.5% in the primary cost-effectiveness analysis (Figure 1B). Using QALYs, the incremental cost-effectiveness ratio point estimate rose from £20,840 to £42,660 per QALY; and the probability that steroid injection is cost-effective decreased to between 33.5% and 43.4% (Figure 2D) in the NICE threshold range of £20,000 to £30,000 per QALY, compared with 49.7% and 54.9% in Figure 1D.

Substituting the FHT-enhanced EQ-5D for the original EQ-5D-3L again brings the incremental cost-effectiveness ratio below the lower UK threshold of £20,000, specifically to £13,800; and increases the probability of cost-effectiveness to 67.4% at the lower threshold of £20,000 and 77.2% at the higher threshold of £30,000 (Figure 2F).

## Discussion

The MortISE trial with 131 participants found that foot health in the steroid group was significantly better than in the control group: the mean difference in the patient-centered Foot Health Thermometer (FHT) at 3 months was 14.1 scale points (95% CI: 5.5 to 22.8 points; p = 0.002) [8]. In the reduced sample of 109 participants for whom we had enough data for economic analysis, the mean difference fell to 11.9 scale points (95% CI: 2.5 to 21.3 points; p = 0.013). When divided by the corresponding standard deviations of the baseline FHT scores, these differences yield ‘effect sizes’ of 0.62 and 0.53 respectively. Effect sizes greater than 0.5 are generally regarded as moderate and therefore worthwhile [30].

This economic evaluation supports and extends the findings from our clinical trial. If decision makers value an improvement of one point on the FHT over one year at £100, the upper confidence limit of the corresponding incremental cost-effectiveness ratio, then the use of ultrasound-guided steroid injection to alleviate foot pain from Morton’s neuroma is cost-effective with a probability of 97.5%; and a cost per point improvement on the FHT of only £32. In contrast, our secondary cost-utility analysis generated a cost per QALY of £20,840 and probability of cost-effectiveness little more than 50% across the recommended UK threshold, which ranges from £20,000 to £30,000 [10]. Though this estimated incremental cost-effectiveness ratio is similar to that for using steroid treatment to manage sciatica [31], the discrepancy between our cost-effectiveness and cost-utility analyses alerted us to the danger that the latter had suffered from the known lack of responsiveness of the original EQ-5D [26,27].

We addressed this discrepancy by a sensitivity analysis that replaced the original EQ-5D by an enhanced version combining the economic validity of the EQ-5D with the greater responsiveness of the FHT. This reduced the incremental cost-effectiveness ratio to £6,400, well below the lower UK threshold of £20,000. This analysis also increased, to more than 80%, the probability that steroid injections for Morton’s neuroma are cost-effective. We believe this analysis, nominally secondary, has bridged the gap between our positive findings about effectiveness [8] and our initially negative findings about cost-effectiveness. The strength of the association in our data between EQ-5D and FHT has convinced us that the strong evidence about effectiveness from the condition-specific FHT translates into sufficient evidence about cost-effectiveness from the enhanced EQ-5D.

Our second sensitivity analysis indicates that, if control participants attend outpatient clinics and receive injection but no ultrasound scan, the incremental cost-effectiveness ratio would rise from £32 to £65 per point improvement in FHT-AUC. This is still below our illustrative cost ceiling of £100. However, the cost per QALY would rise to £42,660, well above the higher UK threshold of £30,000.

As we are reporting findings after three months, we do not know whether the improvement in foot health was maintained, increased or reduced. As we explained in the main effectiveness paper of the MortISE trial [8], it is not appropriate to extrapolate effectiveness or cost-effectiveness findings beyond the period of data collection.

We believe this is the first cost-effectiveness study of steroid injections to alleviate pain from Morton’s neuroma. As we cannot compare our findings with other cost-effectiveness analyses in Morton’s neuroma, we have compared them with steroid treatment in managing sciatica.

The conclusions from this trial suffer from follow-up of only three months and our focus on direct costs to the National Health Service, rather than patient-borne costs. Though we would have preferred longer follow-up to assess the effect of steroid injection on subsequent surgical rates, the ethical committee insisted that we offer the intervention to participants in the control group after three months [8].

Though we asked participants about their own resource use, the self-reported client service receipt inventory is an accepted method of data collection when the recall period is only three months [32]. Furthermore, it facilitates the collection of data from many sources, as in this trial, where we are interested in both primary and secondary healthcare sectors. Hence, we see self- reporting in these patients as efficient use of research resources. For participants referred to private hospitals, we tried to obtain data on the resulting costs, but failed because those hospitals would not give us access to commercially sensitive data.

This trial has shown that steroid injection is effective and cost-effective for the National Health Service in treating Morton’s neuroma in the short term. Any further trial of the management of Morton’s neuroma should plan a longer follow-up period. It could also explore the effectiveness and cost-effectiveness of combinations of treatments, including the provision of insoles, analgesia and physiotherapy as well as steroid injection. It could also adopt a wider perspective to include patient-borne costs of attending hospital for treatment and of self-management of this condition.

## Conclusions

Steroid injections are effective and cost-effective in relieving foot pain measured by the Foot Health Thermometer (FHT) for three months. However cost-utility analysis was initially inconclusive because the EQ-5D-3L is less responsive than the FHT to changes in foot health. By using the FHT to enhance the EQ-5D, we inferred that steroid injections yield good value in cost per QALY.

## Abbreviations

CI: Confidence interval
EQ-5D-3L: European quality of life-5 dimensions–3 levels
FHT: Foot health thermometer
FHT-AUC: Area under the curve of the foot health thermometer
FHT-enhanced EQ-5D: Foot health thermometer-enhanced European Quality of life-5 Dimensions
NICE: National Institute for Health and Care Excellence
QALY: Quality-adjusted life year
